# Supplementary material for: End Sequence Analysis Toolkit (ESAT) expands the extractable information from single-cell RNA-seq data
Source: Genome Res. 2016 Oct;26(10):1397–410. doi: 10.1101/gr.207902.116 (PMC5052061; doi:10.1101/gr.207902.116)
Supplement: Supplemental Material [file supp_gr.207902.116_Supp_Materials.tar.gz › ESAT-esat_v0.1/doc/html/classbroad_1_1pda_1_1rnai_1_1_r_n_ai_gene_annotation.html]

ESAT: broad.pda.rnai.RNAiGeneAnnotation Class Reference


ESAT


- Main Page
- Related Pages
- Packages
- Classes
- Files

- Class List
- Class Index
- Class Hierarchy
- Class Members

All Classes Namespaces Files Functions Variables Enumerator Pages

- broad
- pda
- rnai
- RNAiGeneAnnotation

Public Member Functions |
Static Public Attributes |
List of all members

broad.pda.rnai.RNAiGeneAnnotation Class Reference

Collaboration diagram for broad.pda.rnai.RNAiGeneAnnotation:

[legend]

|  |  |
| --- | --- |
| Public Member Functions | |
|  | RNAiGeneAnnotation (Gene gene, Alignments linc, String geneName, int versionNumber, String seq, String species, String ncbiBuild) |
|  | |
|  | RNAiGeneAnnotation (String rnaiString) |
|  | |
| String | toString () |
|  | |
| String | toRNAi () |
|  | |
| String | getTranscriptSequence () |
|  | |
| String | getTranscriptName () |
|  | |
| Alignments | getRegion () |
|  | |
| String | getGeneName () |
|  | |
| Sequence | getSequence () |
|  | |
| String | toNanostring () |
|  | |

|  |  |
| --- | --- |
| Static Public Attributes | |
| static String | nanostringHeader ="GENE.SOURCEID\tGENE.TAXONID\tGENE.NCBI\_BUILDID\tGENE.SOURCECHR\tGENE.SOURCESTART\tGENE.SOURCEEND\tGENE.SOURCESTRAND\tTRANS.SOURCEID\tTRANS.SEQ\tTRANS.LENGTH" |
|  | |

## Constructor & Destructor Documentation

|  |  |  |  |
| --- | --- | --- | --- |
| broad.pda.rnai.RNAiGeneAnnotation.RNAiGeneAnnotation | ( | Gene | *gene*, |
|  |  | Alignments | *linc*, |
|  |  | String | *geneName*, |
|  |  | int | *versionNumber*, |
|  |  | String | *seq*, |
|  |  | String | *species*, |
|  |  | String | *ncbiBuild* |
|  | ) |  |  |

Here is the call graph for this function:

|  |  |  |  |  |  |
| --- | --- | --- | --- | --- | --- |
| broad.pda.rnai.RNAiGeneAnnotation.RNAiGeneAnnotation | ( | String | *rnaiString* | ) |  |

## Member Function Documentation

|  |  |  |  |  |
| --- | --- | --- | --- | --- |
| String broad.pda.rnai.RNAiGeneAnnotation.getGeneName | ( |  | ) |  |

|  |  |  |  |  |
| --- | --- | --- | --- | --- |
| Alignments broad.pda.rnai.RNAiGeneAnnotation.getRegion | ( |  | ) |  |

|  |  |  |  |  |
| --- | --- | --- | --- | --- |
| Sequence broad.pda.rnai.RNAiGeneAnnotation.getSequence | ( |  | ) |  |

|  |  |  |  |  |
| --- | --- | --- | --- | --- |
| String broad.pda.rnai.RNAiGeneAnnotation.getTranscriptName | ( |  | ) |  |

|  |  |  |  |  |
| --- | --- | --- | --- | --- |
| String broad.pda.rnai.RNAiGeneAnnotation.getTranscriptSequence | ( |  | ) |  |

|  |  |  |  |  |
| --- | --- | --- | --- | --- |
| String broad.pda.rnai.RNAiGeneAnnotation.toNanostring | ( |  | ) |  |

|  |  |  |  |  |
| --- | --- | --- | --- | --- |
| String broad.pda.rnai.RNAiGeneAnnotation.toRNAi | ( |  | ) |  |

Here is the call graph for this function:

|  |  |  |  |  |
| --- | --- | --- | --- | --- |
| String broad.pda.rnai.RNAiGeneAnnotation.toString | ( |  | ) |  |

Here is the caller graph for this function:

## Member Data Documentation

String broad.pda.rnai.RNAiGeneAnnotation.nanostringHeader ="GENE.SOURCEID\tGENE.TAXONID\tGENE.NCBI\_BUILDID\tGENE.SOURCECHR\tGENE.SOURCESTART\tGENE.SOURCEEND\tGENE.SOURCESTRAND\tTRANS.SOURCEID\tTRANS.SEQ\tTRANS.LENGTH"

static

---

The documentation for this class was generated from the following file:

- java/broad/pda/rnai/RNAiGeneAnnotation.java


---

Generated on Fri Aug 15 2014 15:11:01 for ESAT by  

 1.8.7
